# Quantifying rapidly declining abundance of insects in Europe using a paired experimental design

**DOI:** 10.1002/ece3.6070

**Published:** 2020-02-12

**Authors:** Anders Pape Møller

**Affiliations:** ^1^ Ministry of Education Key Laboratory for Biodiversity Science and Ecological Engineering College of Life Sciences Beijing Normal University Beijing China; ^2^ Ecologie Systématique Evolution Université Paris‐Sud CNRS AgroParisTech Université Paris‐Saclay Orsay Cedex France

**Keywords:** declining abundance, insects, land‐use, latitudinal decline

## Abstract

The abundance of insects has decreased for the last decades in many parts of the world although so far few studies have quantified this reduction because there have only been few baseline studies dating back decades that have allowed comparison of ancient and recent population estimates. Such a paired design is particularly powerful because it reduces or eliminates bias caused by differences in identity and experience of observers, identity of study sites, years, time of season, and time of day, and it ensures identity of sampling procedures. Here, I compiled information on the reduction in abundance of insects in Europe and Algeria by the same persons compiling the abundance of insects from the same 21 study sites during 1951–1997 and again a second time in 1998–2018. There was a reduction by 47% in the abundance of insects. The difference in abundance in old compared to new samples declined with latitude, with a significant variance among taxa. This reduction in abundance of insects was of such a magnitude that it must have consequences for insectivores and the role that insects play in ecosystems.

## Graphical abstract

Insects in Europe have decreased in abundance by more than 70% since the mid 1960s in paired collections in the same sites in the 1960s and in 2015–2019.

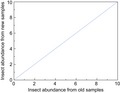

## 1 INTRODUCTION

Extensive surveys of insects have shown dramatic reductions in abundance by as much as 80%, even in nature reserves (Hallmann et al., 2017; Møller, 2019; Morrissey et al., 2015; Sánchez‐Bayo & Wyckhuys, 2019). These changes have been attributed to a diversity of drivers including farming practice, land‐use, and the associated factors such as use of pesticides, biological interactions, and climate change (Sánchez‐Bayo & Wyckhuys, 2019; Vogel, 2017). While some studies have documented such reductions in insect abundance over time (Hallmann et al., 2017; Sánchez‐Bayo & Wyckhuys, 2019), others have shown little or no change in abundance of insects (Conrad, Warren, Fox, Parsons, & Woiwod, 2006; Conrad, Woiwod, & Perry, 2002; Shortall et al., 2009). This raises questions about generality, but also the underlying mechanisms accounting for heterogeneity in such effects. In other words, which are the factors that account for these reductions in abundance, and do taxa differ in their reduction in abundance in a predictable way.

Biodiversity and abundance generally show latitudinal gradients although the causes of such differences among taxa are poorly known (Ball‐Damerow, M'Gonigle, & Resh, 2014; Deutsch et al., 2008; Jacobson, Tucker, Mathiasson, & Rehan, 2018; Rohde, 1992; Tierno de Figueroa et al., 2010). Although a number of hypotheses have been proposed to account for such gradients, differences in life history such as generation time and differences in the relative importance of interspecific interactions have been hypothesized to be particularly important. Likewise, climate change has caused a strong decline in abundance of many taxa including insects (Parmesan & Yohe, 2003; Root et al., 2003). Recent dramatic changes in climatic conditions have been shown experimentally to affect normal sperm function in insects (Sales et al., 2018). Therefore, I investigate the extent to which latitude and taxa affected the decline in abundance of insects. Because farming practices and land‐use have changed recently (Møller, 1980, 1983, 2019; Sánchez‐Bayo & Wyckhuys, 2019), this may greatly have impacted insect abundance since just a small amount of remaining pristine habitats is outside the reach of the decrease in diversity and abundance of insects in farmland. Changes in land‐use by plants may drastically reduce the biomass of caterpillars when invasive plants invade hedgerows (Richard, Tallamy, & Mitchell, 2018).

Although there are several ways in which quantitative insect surveys can cause bias and even systematic bias over time (Møller, 2019; Sánchez‐Bayo & Wyckhuys, 2019), there have been few attempts to quantify such bias (The Economist, 2019). The ideal procedure for tests of population trends depends on several strict criteria that reduce or minimize biased estimates. These include (a) surveys in the same study site in the first and the second year, (b) surveys conducted by the same person in the first and the second year, (c) surveys conducted at the same time of the season, (d) surveys conducted at the same time of the day, and (e) surveys conducted with the same method in the first and the second year. The larger the number of matched survey criteria, the smaller the risk of bias in survey results. Here, I attempt to restrict tests for reductions in the abundance and diversity of insects by using comparisons between such matched pairs of surveys.

The objectives of this study were to (a) test whether the abundance of insects has decreased during the recent three decades relying on studies where closely matched samples allowed for comparison across long temporal scales, (b) whether this decline differed among habitats, and (c) whether the decline differed among latitudes. To this end, I collected information from recent published and unpublished studies and combined this information with published and unpublished records covering the same study areas and study periods by the same scientists. This paired study method constitutes a particularly powerful method for quantifying the decline in insect abundance while controlling for potentially confounding variables.

## 2 METHODS

The main study sites in Denmark were at Kraghede (57°N, 10°00°E), where open farmland habitats were surveyed extensively for insects in 1970–1974 and again in 2015–2018. The main crops in this agricultural landscape were grass, potatoes, wheat, beets and to a smaller extent barley, oats, rye, and other crops. Less intensely cultivated or uncultivated habitats were streams, ditches, ponds, road verges, hedgerows, and plantations. Changes in the extent of these natural habitats and other components of land‐use are shown in Møller (1980),

The present study of 45 km^2^ farmland areas in Northern Denmark was part of an initiative for studying the effects of intensified farm land‐use and farming practices on diversity and abundance of insects and birds (Møller, 1980, 1983).

Old studies that were originally made around 1970 were repeated in 2015–2018 in the same study plots by the same scientist as originally in order to ensure that there was no heterogeneity due to differences in study sites and among observers. These 18 study plots were combined with seven published data sets (reported in Conrad et al., 2006; Conrad et al., 2002; Hallmann et al., 2017; Hofmann, Fleischmann, & Renner, 2018; Møller, 2019; Schuch, Bock, Krause, Wesche, & Schaefer, 2012; Shortall et al., 2009) that were similarly investigated twice by the same scientists during an early study in 1970–1975 and again in 2015–2018.

I used the following procedures for quantifying the abundance of insects. I counted the number of dead insects on the windscreen of a car driven at a fixed speed of 80 km, while recording the area of the windscreen, weather, and the brand of car (Møller, 2013). This was done at the same road for 41 25 km transects in 1997 and again for 41 transects in May–August 2017 at Pandrup, Denmark. Likewise, 47 transects of 10 km were made in May–August 1997 and 33 transects during the same period at Badajoz, Spain. Finally, I made 76 transects of 2 km during May–August 1997 and 62 transects during May–August 2017 at Kraghede, Denmark. Studies of insects killed by windshields on a car may be biased if the aerodynamics and the size of cars changes systematically. A number of methodological tests have investigated such potential bias (Møller, 2013, 2019, unpublished). These studies have shown a high degree of consistency among sampling methods (windshields, sweep‐nets, feeding rates of barn swallow nestlings, sticky traps, and samples of insects derived from windshields and the sound when large insects have an impact with the windshield (Møller, 2013, 2019, unpublished). Finally, it is possible to include car brand as a random factor in a statistical model and thereby adjust for differences in sampling effort among car brands. Such analyses only showed weak effects of car brand on insect abundance (Møller, 2019).

I made surveys of the number of carabid beetles in straw carts on 10 different days in 10 different farms in August 1970 and again in the same farms in August 2017 counting the number of beetles after the straw had been removed by the farmers.

I used sweep‐nets for catching butterflies on 20 days in May–August along a dirt road of 1.4 km in 1970 and again along the same dirt road in May–August 2017 at Kraghede, Denmark. This road is bordered by verges.

I used a sweep‐net with a diameter of 1 m to collect insects at a height of 1.5 m and a maximum distance of 100 m from breeding sites of barn swallows *Hirundo rustica*. The number of sweeps was standardized to one per 10 s while walking at a speed of 5 km per hour. All sweeps were made from 10:00 to 14:00 at the time of the day when insects are most abundant. Barn swallows spend most of their foraging time within a distance of 100 m from their breeding sites (Møller, 1987). These samples were collected in farmland surrounding 23 farms at Kraghede, Denmark in June–July 1984 and on the same 23 farms in June–July 2017 (Møller, 1987). Insect abundance was estimated as the number of individuals at the end of a transect.

Barn swallows mainly feed on insects living on middens and in farms with domestic animals, mainly cattle. Because barn swallows mainly feed on insects within a distance of 100 from farms with distances up to 500 m (Møller, 1987, 2001), William Carøe Årestrup and I obtained estimates of insects on middens at 30 farms around Pandrup, Denmark, in June–July 1970 and again on the same farms in June–July 2017 on a log scale from 1, 10, 100, 1,000, 10,000, 100,000 to 1,000,000. We made a similar survey at 50 farms at Kraghede, Denmark, 1970, and again at the same farms in 2017.

Finally, I estimated the height of anthills constructed by *Formica rufa* and measured to the maximum height with a ruler to the nearest cm in small conifer plantations at Kraghede, Denmark, in May–June 1971 and again at the same plantations in May–June 2017. This survey was part of an extensive survey of breeding birds in these study areas (Møller, 1991).

In addition to the insect samples described above. I used published data on insect abundance that had been collected in 1951–1975 and again in 2015–2018 by Benslimane, Chakri, Haiahem, Samraoui, and Samraou (2018), Conrad et al. (2002), Conrad et al. (2006), Hofmann et al. (2018) and Hallmann et al. (2017), Schuch et al. (2012) and Shortall et al. (2009). This sampling procedure allowed for estimates of the reduction in insect abundance across long time scales.

I used GLM with normally distributed data and an identity link function to analyze the data. Analyses were weighted by sample size to take differences in abundance among analyses into account. I estimated chance in insect abundance by dividing log_10_‐transformed abundance of insects in the early period by log_10_‐transformed abundance of insects in the recent study period. I included latitude longitude and insect taxon in the analyses (listed in Table S1). I used SAS (2012) for the statistical analyses.

## 3 RESULTS

### 3.1 Total sample sizes

The total abundance of insects for the old surveys was on average 473.15, *SE* = 11.89, *N* = 41 samples, which resulted in a total abundance of 19,309.15, *SE* = 0.53. For the recent surveys, mean value was 11.89, *SE* = 2.89, *N* = 41 samples, which resulted in a total abundance of 487.49, *SE* = 0.17, *N* = 41 samples. In other words, mean reduction between old and recent samples was (19,309.15/(19,309.15 + 473.15))*100, or a reduction by 97.6%.

### 3.2 Paired and old and recent surveys of insects

F. de Lope surveyed insects using the number of insects killed on the windshield on a single major 10 km road at Badajoz, Spain, in spring and summer 1997 and 2018. The mean number of killed insects in 1997 was 34.6 (*SE* = 5.3, *N* = 38 transects), while it was 74.6 (*SE* = 9.0, *N* = 33) in 2018.

I surveyed carabid beetles in 1970–1979 and again in 1980–2017 at Kraghede, Denmark. While counts of carabid beetles on average was 313.8 (*SE* = 35.45, *N* = 10) in 1970–1979, it was only 18.4 (*SE* = 4.7, *N* = 38) in 1980–2017.

I used standard sweep‐net captures at a 1 km stretch at Kraghede, Denmark, in 1970 which revealed 10.7 butterflies (*SE* = 1.3, *N* = 20), and at the same stretch in 2017 a much smaller number of 0.3 butterflies (*SE* = 0.1, *N* = 20).

The number of Diptera om middens at Kraghede, Denmark, in 1970 was on average 1.2 in log_10_‐units (*SE* = 0.04, *N* = 50), while it was only 0.2 (*SE* = 0.2, *N* = 50) in 2017. A similar survey in 1970 at Pandrup, Denmark, by A. P. Møller and W. C. Årestrup was 3.9 in log_10_‐units (*SE* = 0.3, *N* = 50), while a repeated survey in 2017 revealed a mean value of 2.4 (*SE* = 0.3, *N* = 50).

The number of insects caught with sweep‐nets at 23 farms in 1984 was on average 0.7 in log_10_‐units (*SE* = 0.05), while a repeated survey in 2011 revealed a mean value of 0.08 (*SE* = 0.01, *N* = 23).

The height of mounds constructed by *Formica rufa* in 1971 was on average 109.3 cm (*SE* = 4.5, *N* = 30), while it was only 46.5 cm (*SE* = 3.5, *N* = 30) in 2017. All other surveys and the results are reported in the references listed in Table S1.

### 3.3 Meta‐analysis of change in abundance of insects

**Figure 1:**
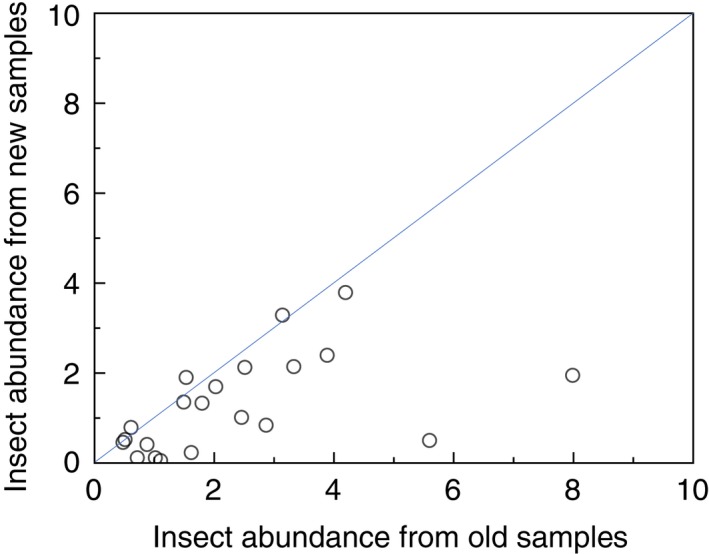
Mean insect abundance in recent samples in relation to mean insect abundance in old and recent samples of insects from the same locations. See the main text for definitions of abundance. The blue line is Y = X. Data are located in Table S1

**Figure 2:**
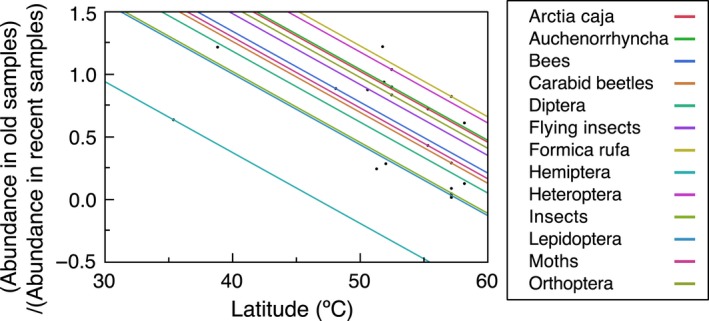
Abundance of insects in old samples relative to the abundance in recent insect samples at the same 21 sites collected by the same persons. Lines of different color show relationships for different taxa at different latitudes based on the statistical model in Table 1

I compiled information on abundance of insects from the same 21 study sites by the same persons in Europe and Algeria (one study site) during an early year in the period 1951–1997 and a late year in the period 1998–2018 (Figure 1). The mean year for the first data compilation was 1976 *SE* = 3.3 years, and again for the second data compilation in 2011, *SE* = 1.5 years, or 36 years after the first insect survey, *SE* = 3.0 years later. Reduction in insect abundance was log abundance in 1976 divided by log abundance in 2011 (Figure 2), which at mean (*SE*) was 0.537 (*SE* = 0.084), statistically significant at *t* = 6.437, *df* = 20, *p* < .0001.

The ratio of log‐transformed abundance of insects in old minus log‐transformed abundance of insects in recent samples was 0.537, *SE* = 0.084, *t* = 6.43, *df* = 20, *p* < .0001). Thus, there was a significant reduction by a factor 3.443 between the two sets of samples.

Abundance in old compared to new samples declined with latitude showing a particularly strong decline in abundance at low latitude with a significant variance among taxa (Table 1). The mean reduction in abundance was 47%, *SE* = 8%, *t* = 5.69, *df* = 20, *p* < .0001. There was a significant difference in decrease in abundance among taxa (Figure 2), but also a significant decrease with increasing latitude (Figure 2). The reduction in abundance was similar for all taxa as shown by a negative slope that did not differ among taxa (Figure 2). The difference in intercept is revealed by the lines in Figure 2 varying in distance among each other (Figure 2). The black dots show the positions for the 18 lines shown in Figure 2. The ratio of abundance of old minus recent samples was 0.554 [95% CI = 0.273, 0.835] at a latitude of 58.22 ºN, while it was 1.866 [95% CI = 1.398, 2.332] at a latitude of 35.42°N.

**Table 1:** Abundance of insects in old samples relative to the abundance of insects in recent insect samples at the same sites collected at 21 site years by the same persons

Source	*df*	LR *χ* ^2^	*p*	Estimate	*SE*
Intercept	1	25.76	.0017	3.652	0.513
Latitude	1	20.73	<.001	−0.058	0.010
Taxon	12	24.75	.016		

Note

The model had the likelihood ratio (LR) statistics *χ* ^2^ = 33.12, *df* = 13, *p* = .0016.

A comparison of the change in log‐transformed abundance of insects in surveys that involved assessment of change in insectivores or not revealed a significantly stronger decline in surveys with insectivores (mean (*SE*) = 0.699 (0.171), *N* = 7) than in surveys without insectivores (0.281 (0.081), *N* = 14; *χ* ^2^ = 6.08, *p* = .014, estimate (*SE*) = −0.209 (0.079)).

## 4 DISCUSSION

The abundance of insects has decreased considerably in many areas across the world (review in Sánchez‐Bayo & Wyckhuys, 2019). For example, Hallmann et al. (2017) have documented declines in insect abundance in nature reserves in Germany by 80%. Here, I have compiled 21 sets of paired samples of insects that were collected by the same persons in the same study sites and with the same sampling procedures on average in 1976 and again on average in 2011. This paired study method is particularly powerful for quantifying whether there is a systematic decline in insect abundance while controlling for potentially confounding variables. I am unaware of any other study using a similar rigorous approach (e.g., the review by Sánchez‐Bayo and Wyckhuys (2019) did not provide other similar studies).

The general cause of these dramatic reductions in insect abundance remains poorly understood, although Sánchez‐Bayo and Wyckhuys (2019) have made an exhaustive compilation of potential drivers that included land‐use, pesticides, biological factors that include parasites and pathogens and climate change. Several studies have suggested that an important cause for such declines of insects is intensified land‐use (Fox et al., 2014; Møller, 1980, 1983, 2001; Sánchez‐Bayo & Wyckhuys, 2019), while others have emphasized the importance of pesticide use (Hallmann, Foppen, Turnhout, Kroon, & Jongejans, 2014; Hofmann et al., 2018; Møller, 1980, 1983; Pomfret, Nocera, Kyser, & Reudink, 2000; Sánchez‐Bayo & Wyckhuys, 2019).

Land‐use in the study site at Kraghede, Denmark, that accounted for most of the datasets reported here, has been relatively modest with 80%–90% of crops being grass, potatoes, wheat, and beets. Detailed information on land‐use including ditches, ponds, and plantations are provided in detail in Møller (1983).

Insect abundance has been shown to vary with latitude in a number of studies although differences in sampling method and heterogeneity in sampling procedures among observers were never or rarely considered (Rohde, 1992). Here, I have documented a latitudinal change in abundance of insects. At higher latitude, there was a similar abundance of insects in 1980 as in 2012, while at low latitudes there was a clearly increasing difference. The present study varied in latitude from 35.4°N to 58.2°N, mean (*SE*) 52.7°N (1.3), *N* = 18. It is interesting to notice that declines in the abundance of aerial insectivorous birds have followed a similar latitudinal decline as shown for insect abundance (Nebel, Mills, McCracken, & Taylor, 2010). This is consistent with insect abundance driving changes in the abundance of insectivores and not the reverse.

Pesticides have been suggested to play a crucial role in controlling the abundance or resulting in a decline in insect abundance in recent years (Fox et al., 2014; Hallmann et al., 2014; Nocera et al., 2012; Poulin, Lefebvre, & Paz, 2010; Sánchez‐Bayo & Wyckhuys, 2019). This decline varied among insect taxa. I am still unaware of any large‐scale studies experimentally testing for such effects.

Insects are consumed by insectivores, and reductions in population size of insects result in matching reductions in the number of insectivores. Indeed, there is a tight positive association between population size of insectivorous birds and population size of insects (Møller, 2019).

The findings reported here must have severe implications for insectivores such as many birds (Hallmann et al., 2014; Møller, 2019), but also for pollination and other biological interactions (Sánchez‐Bayo & Wyckhuys, 2019). Indeed, avian insectivores have been shown to closely track the abundance of insects in Danish farmland during a long‐term study of 23 years (Møller, 2019). It is no surprise that many plants with a main distribution in farmland have shown dramatic reductions in diversity and abundance.

## CONFLICT OF INTEREST

The author declares no competing interests.

## AUTHORS CONTRIBUTIONS

The author was responsible for the entire study.

## ETHICAL APPROVAL

Collection of insect samples did not require ethics approval.

## Data Availability

All data are available in Table S1 in this paper.
