# Supplementary material for: Quantifying rapidly declining abundance of insects in Europe using a paired experimental design
Source: Ecol Evol. 2020 Feb 12;10(5):2446–51. doi: 10.1002/ece3.6070 (PMC7069279; doi:10.1002/ece3.6070)
Supplement: Supplementary file 1 [file ECE3-10-2446-s001.docx]

**Table S1**. Insect abundance for different taxa in 21 site years, their coordinates, survey methods, year, mean abundance, SE abundance and sample size for first surveys (year 1, sample size is N 1) and last surveys (year 2, sample size is N 2). N 1 and N 2 are the number of samples collected in each study year.

| Taxon | Habitat | Locality | Latitude (ºN) | Longitude (ºE) | Survey method | Year 1 | Abundance 1 | SE 1 | N 1 | Year 2 | Abundance 2 | SE 2 | N 2 | Reference |
| --- | --- | --- | --- | --- | --- | --- | --- | --- | --- | --- | --- | --- | --- | --- |
| Insects | Farmland | Pandrup, Denmark | 58.22 | 9.67 | Car transect | 1997 | 1.63 | 0.68 | 41 | 2017 | 0.20 | 0.01 | 41 | Møller (2019) |
| Insects | Farmland | Badajoz, Spain | 38.88 | -6.97 | Car transect | 1997 | 1.55 | 0.80 | 47 | 2018 | 1.87 | 0.96 | 33 | F. de Lope unpublished |
|  |  |  |  |  |  |  |  |  |  |  |  |  |  |  |
| Carabid beetles | Farmland | Kraghede, Denmark | 57.20 | 10.07 | In farm cart | 1970 | 2.50 | 1.54 | 10 | 2017 | 0.72 | 0.49 | 10 | A. P. Møller unpublished |
| Lepidoptera | Farmland | Kraghede, Denmark | 57.20 | 10.07 | Sweep-net | 1970 | 1.30 | 0.04 | 20 | 2017 | 0.08 | 0.03 | 20 | A. P. Møller unpublished |
| Diptera | Farmland | Kraghede, Denmark | 57.20 | 10.07 | Counts on middens | 1970 | 1.12 | 0.04 | 50 | 2017 | 0.02 | 0.20 | 50 | A. P. Møller unpublished |
| Hemiptera | Other | Numidia, Algeria | 35.42 | 6.26 | Sweep-net | 1996 | 3.34 |  | 24 | 2012 | 2.11 |  | 24 | N. Benslimane unpublished |
| Diptera | Farmland | Pandrup, Denmark | 58.22 | 9.67 | Counts on middens | 1970 | 3.90 | 0.29 | 30 | 2017 | 2.37 | 0.26 | 30 | W. C. Aarestrup unpublished |
| Diptera | Farmland | Kraghede, Denmark | 57.20 | 10.07 | Sweep-net | 1984 | 0.73 | 0.05 | 23 | 2017 | 0.08 | 0.01 | 23 | A. P. Møller unpublished |
| Insects | Other | Krefeld, Germany | 51.34 | 6.59 | Malaise traps | 1990 | 8.00 |  | 96 | 2017 | 1.92 |  | 96 | Hellmann et al. (2017) |
| Insects | Farmland | Kraghede, Denmark | 57.20 | 10.07 | Car transect | 1997 | 5.61 | 0.68 | 76 | 2017 | 0.47 | 0.11 | 62 | Møller (2019) |
| Moths | Farmland | UK | 55.36 | -3.44 | Traps | 1967 | 0.89 |  | 100 | 2003 | 0.38 |  | 100 | Conrad et al. (2006) |
| *Arctia caja* | Farmland | UK | 55.36 | -3.44 | Light trap | 1968 | 1.81 |  | 56 | 1998 | 1.30 |  | 70 | Conrad et al. (2002) |
| Flying insects | Farmland | Hereford, UK | 52.06 | -2.72 | Suction trap | 1973 | 2.88 |  | 26 | 2002 | 0.81 |  | 26 | Shortall et al. (2009) |
| Flying insects | Farmland | Rothamstead, UK | 51.81 | -0.36 | Suction trap | 1973 | 0.62 |  | 26 | 2002 | 0.76 |  | 26 | Shortall et al. (2009) |
| Flying insects | Farmland | Starcross, UK | 50.63 | -3.45 | Suction trap | 1973 | 0.49 |  | 26 | 2002 | 0.43 |  | 26 | Shortall et al. (2009) |
| Flying insects | Farmland | Wye, UK | 51.92 | -2.34 | Suction trap | 1973 | 0.53 |  | 26 | 2002 | 0.49 |  | 26 | Shortall et al. (2009) |
| Bees | Other | Munich, Germany | 48.14 | 11.58 | Sweep-net | 1997 | 1.50 | 0.11 | 123 | 2017 | 1.33 | 0.11 | 123 | Hoffman et al. (2018) |
| *Formica rufa* | Farmland | Kraghede, Denmark | 57.20 | 10.07 | Mound height (cm) | 1971 | 2.04 | 0.66 | 30 | 2017 | 1.67 | 0.55 | 30 | A. P. Møller unpublished |
| Auchenorhyncha | Farmland | Stolzenau | 52.52 | 9.07 | Sweep-net | 1951 | 4.21 |  | 9 | 2009 | 3.76 |  | 9 | Schuch et al. (2012) |
| Heteroptera | Farmland | Stolzenau | 52.52 | 9.07 | Sweep-net | 1951 | 3.15 |  | 9 | 2009 | 3.26 |  | 9 | Schuch et al. (2012) |
| Orthoptera | Farmland | Stolzemau | 52.52 | 9.07 | Sweep-net | 1951 | 2.53 |  | 9 | 2009 | 2.10 |  | 9 | Schuch et al. (2012) |
|  |  |  |  |  |  |  |  |  |  |  |  |  |  |  |
